# The life of [*PSI*]

**DOI:** 10.1007/s00294-017-0714-7

**Published:** 2017-06-26

**Authors:** Brian Cox, Mick Tuite

**Affiliations:** 1 Kent Fungal Group, School of Biosciences, University of Kent, Canterbury, Kent CT2 7NJ UK; 2 Linacre College, Oxford University, St. Cross Rd, Oxford, OX1 3JA UK

**Keywords:** Prion, [PSI], Sup35, Hsp104, Sporulation, Yeast

## Abstract

The AAA+ disaggregase Hsp104 is essential for the maintenance and inheritance of nearly all known prions of the yeast *Saccharomyces cerevisiae*. Uniquely for [*PSI* ^+^], the prion form of the Sup35 protein, there seem to be two activities, involving differing co-chaperones, by which Hsp104 affects the inheritance of [*PSI* ^+^], the prion form of the Sup35 protein. Each pathway is also involved in protection against ageing, one through disaggregation of damaged proteins and the other through their retention in the mother cell during budding. Mutations in both Hsp104 and Sup35 affect prion inheritance by one or other of these pathways, as does manipulation of either Hsp104 enzyme activity or expression, in both vegetative (budding) divisions and in sporulation. Based on our recent finding (Ness et al. in Molec Microbiol 104:125–143, 2017) we suggest that the management of the heritable prion forms of Sup35 in [*PSI* ^+^] cells in sporulation may be a marker for a role for Hsp104 in rejuvenation during sporulation.

## Introduction

Prions are proteins which can adopt various forms of aggregation and folding which affect the phenotype of the ‘host’ organism and are heritable or infectious. Commonly cited models are the neurodegenerative diseases in mammals linked to the prion forms of the PrP protein expressed in the brain (Prusiner 2013, review), in *E*. *coli*, a protein, *curli*, which is excreted and helps in the formation of biofilms and in the yeast *Saccharomyces cerevisiae*, the [*PSI* ^+^] prion, which is an aggregate of the polypeptide chain termination factor Sup35/eRF3 (Tuite et al. 2015, review). In *S*. *cerevisiae*, the prion-associated phenotypes are stable over thousands of cell generations, while PrP-based infections are essentially incurable.

**Fig. 1:**
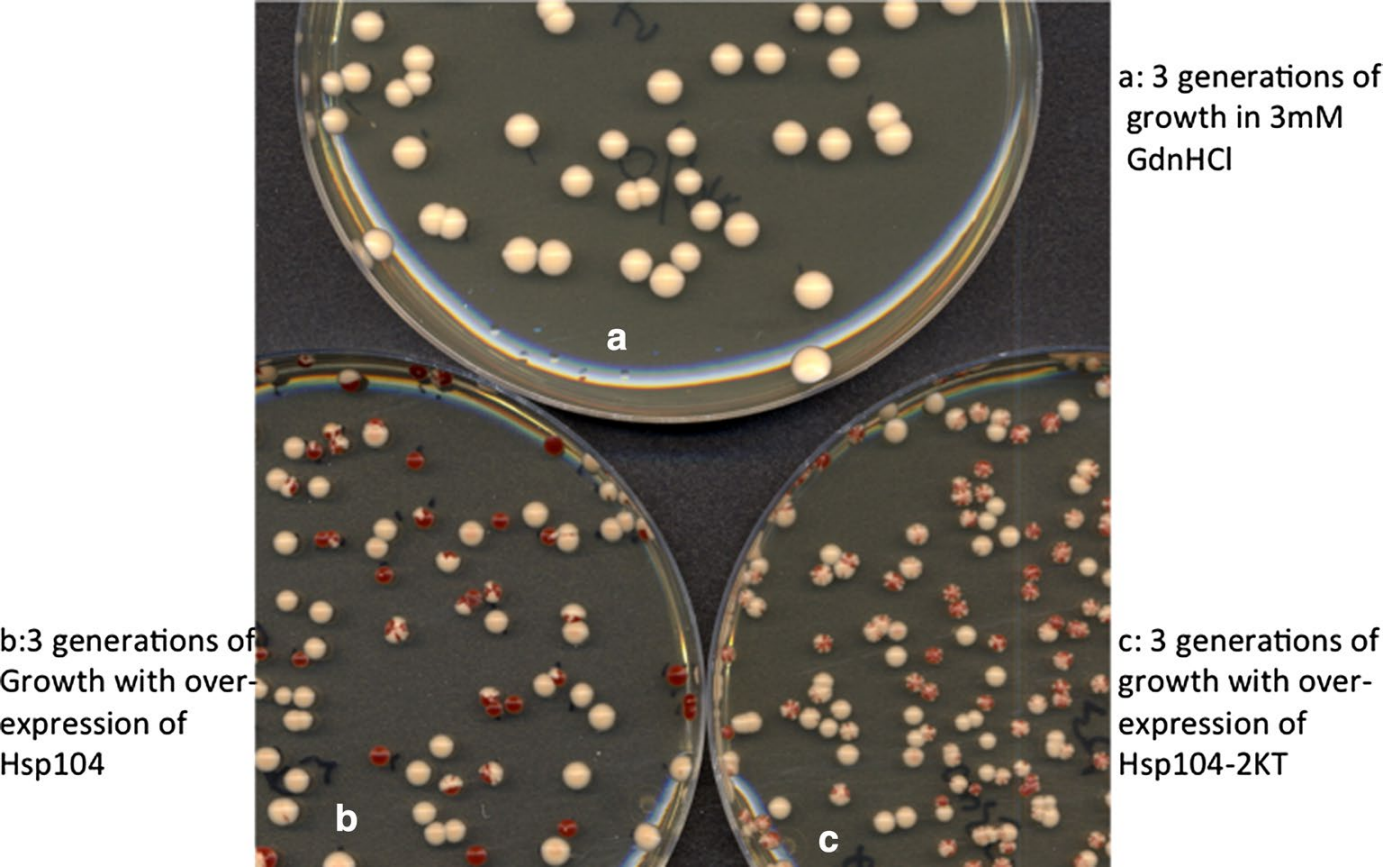
Three modes of curing the [*PSI* ^+^] prion. Plating cultures of the 74D-694 [*PSI* ^+^] strain grown for three generations in 5 mM GdnHCl (*top*) or over-expressing Hsp104 (*bottom left*) or Hsp104^K218T,K620T^ (*bottom right*). In this experiment 22% of the colonies *bottom left* were wholly red (i.e. [*psi* ^−^]) and the remainder either wholly *white* [*PSI* ^+^] or *half red* and *half white* having segregated [*PSI* ^+^] and [*psi* ^−^] daughters in the first division after plating onto the agar. In contrast, every colony from cells over-expressing Hsp104^K218T, K620T^, like those in process of curing by GdnHCl (*top*) must have started from a cell carrying at least one [*PSI* ^+^] propagon because each colony has one (often more) white [*PSI* ^+^] sectors of variable numbers and sizes, indicating that it was genetically [*PSI* ^+^] at the time of plating onto agar. No *red* [*psi* ^−^] colonies were observed. Refer to Fig. 2 for the percentages of [*PSI* ^+^] colonies at this time point

Most, if not all prions so far identified consist of amyloid fibres in which the individual proteins, unlike the native form, are rich in β-sheet. The phenotype which is most commonly used to identify [*PSI* ^+^] strains of yeast arises through a deficiency in chain termination at nonsense codons. The indicator read-through phenotype is an alleviation of nonsense mutations in vitro or in vivo in either the *ADE1* or the *ADE2* gene (Cox 1965; Tuite et al. 1983). Such mutations cause a red pigment to accumulate in cells; the [*PSI* ^+^] prion prevents that and colonies of [*PSI* ^+^] *ade1*-*14* mutants, for example, are white (Fig. 1). From the earliest days of its discovery (Cox 1965), the colour difference between [*PSI* ^+^] and [*psi* ^−^] strains has allowed conditions in which the inheritance of the prion is rendered unstable, to be readily identified.

**Fig. 2:**
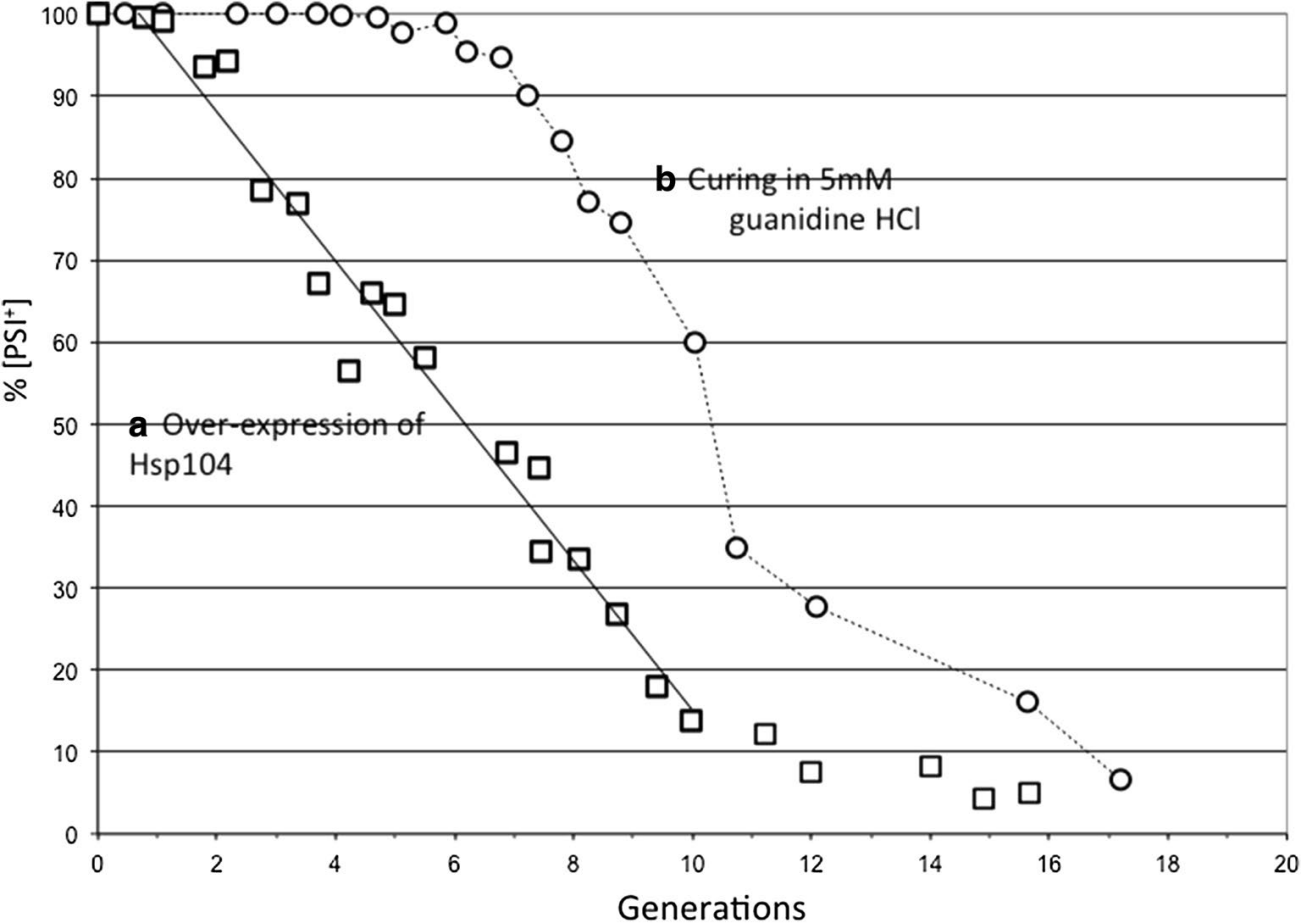
Two modes of [*PSI* ^+^] elimination involving Hsp104. Elimination of [*PSI* ^+^] by over-expression of Hsp104 (*empty square*) shows very different kinetics to elimination by inhibition of Hsp104 ATPase by adding 5 mM guanidine hydrochloride to the medium (*empty circle*). In this experiment Hsp104 over-expression was driven from the *GAL1, 10* promoter

Both inheritance and infection of prions are the consequence of the ability of amyloid fibre to template the addition and refolding of monomeric protein to the growing fibres with fragmentation increasing their numbers. The remodeling and addition of molecules to an aggregate can occur spontaneously, but is greatly enhanced by pre-existing remodeled molecules. The new additions adopt the form of the model template, and fragmentation promotes an exponential increase in amount of amyloid from a pool of monomer both in vitro and in vivo.

A number of factors have been found to affect the stability of inheritance of prions. The stability of [*PSI* ^+^] is affected by environmental stress (e.g. Singh et al. 1979; Tuite et al. 1981; Newnam et al. 2011) or by mutations in either the *SUP35* or the *HSP104* gene (Young and Cox 1971; Doel et al. 1994; DePace et al. 1998) or by manipulation of gene expression (Chernoff et al. 1995; Glover and Lum 2009; Helsen and Glover 2012a, b; Chernova et al. 2017; Ness et al. 2017). Some of the variant forms of the prion amyloid also show an inherent instability during cell division (Uptain et al. 2001). The degree of instability can range from total loss of the prion (often referred to as ‘curing’) to stochastic random loss characteristically giving rise to red sectors on white [*PSI* ^+^] colonies. The involvement of Hsp104 is because this chaperone is required for the fragmentation of amyloid fibres of nearly all yeast prions so far identified, including the Sup35p [*PSI* ^+^] prion.

## The Hsp104 ‘curing’ paradox

Recently, we provided evidence that definitively supports one of two theories which have been offered for a long-running paradox surrounding the inheritance of [*PSI* ^+^] (Ness et al. 2017). The paradox is as follows: the AAA+ disaggregase Hsp104 is essential for [*PSI* ^+^] inheritance and blocking the ATPase activity of Hsp104 using guanidine hydrochloride (GdnHCl) allows the prion to be diluted out during cell division due to the loss of fragmentation by this disaggregase (Eaglestone et al. 2000; Byrne et al. 2007). But [*PSI* ^+^] can also be eliminated from cells by elevating the levels of the same enzyme (Chernoff et al. 1995). The most straight-forward explanation for this oddity, first offered by Paushkin et al. (1996), was that over-expression caused an increase in the disaggregase activity of the enzyme such that the amyloid aggregates of the prion form of Sup35 were resolved into its constituent monomers in their native form. This would result in the restoration of normal polypeptide chain termination activity to Sup35 and abolishing both the read-through of nonsense codons characteristic of the presence of [*PSI* ^+^] and the templating activity of the prion form of the protein on which its inheritance depends. Yet this hypothesis has now been challenged by our new findings (Ness et al. 2017).

**Fig. 3:**
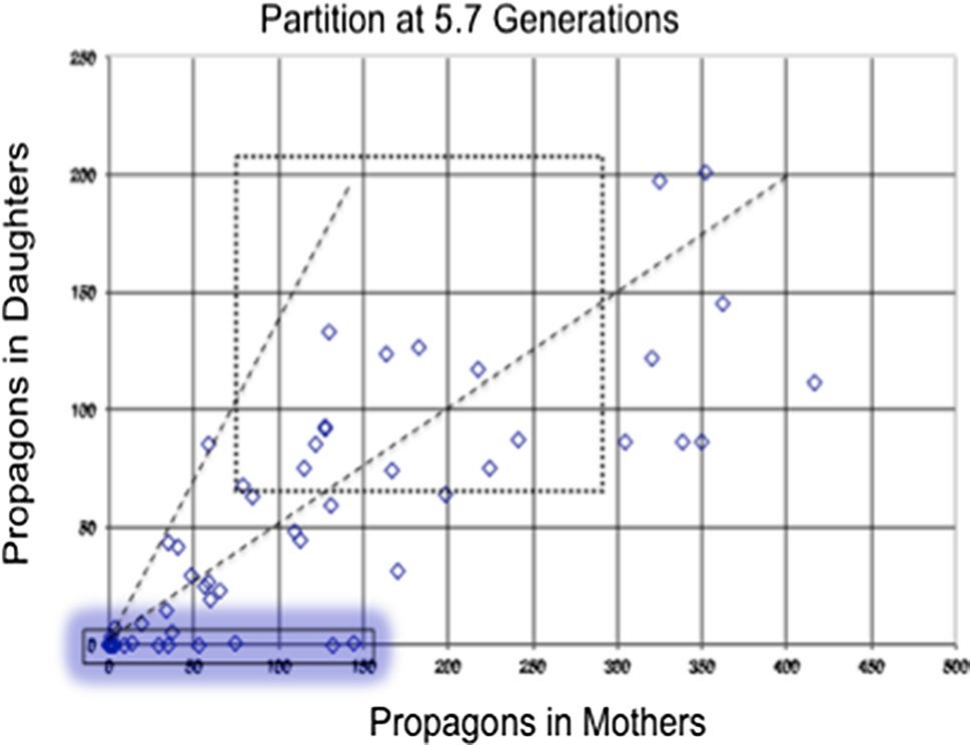
The distribution of [*PSI* ^+^] propagons between mothers and daughters following over-expression of Hsp104. After 5.7 generations post induction of elevated levels of Hsp104, propagon numbers were counted by the single-cell method of Cox et al. (2003) and the numbers in daughter cells are plotted against those in their mothers. The *dotted line box* indicates the limits of such plots in various control and *t*_0_ populations, and the *dashed regression lines* are the observed limits of the proportion of propagons segregating to daughter cells in these populations. The average value of this proportion is 0.4, the approximate ratio of the volumes of mother and daughter cells at cytokinesis (Byrne et al. 2009). The extreme ratios observed in control and *t*_0_ divisions were 0.57 and 0.33. The points below the lower regression are the result of retention of propagons in mother cells. Those *points* indicating divisions where the daughters were [*psi* ^−^] (no popagons) are highlighted

We show that there is no significant degradation of Sup35 [*PSI* ^+^] high molecular weight (HMW) aggregates into lower molecular weight forms when Hsp104 is over-expressed. Rather what happens in many cell divisions is that the genetic units of prion inheritance, entities we call propagons (Cox et al. 2003), are partitioned unequally prior to cells dividing. Retention by the mother cell is such that in about 10% of divisions per generation in our strains and conditions, a daughter cell, and *always* the daughter cell, segregates without any prions and is [*psi* ^−^] (Figs. 1, 3) (Ness et al. 2017). By contrast, the over-expression of an ATPase-negative mutant of Hsp104 (Hsp104^2KT^) in a [*PSI* ^+^] cell leads to the competitive inhibition of the wild type ATPase and the resulting prion loss mimics quantitatively the kinetics of GdnHCl-induced [*PSI* ^+^] curing (Fig. 2) (Ness et al. 2017). However, this mode of curing by Hsp104^2KT^ over-expression, as with GdnHCl-induced curing, results from dilution out of propagons during cell division (Byrne et al. 2007) with a halving in the number of propagons in each generation (Cox et al. 2003; Ness et al. 2017). Nevertheless, the consequences for prion inheritance of over-expression of this mutant differs dramatically from GdnHCl inhibition of Hsp104, in that from the very earliest stages of over-expression red sectors start to appear on the mature colonies growing from [*PSI* ^+^] cells after plating on standard growth medium relieves the over-expression (Fig. 1c). This is a clear indication that over-expression of the mutant Hsp104 induces a change which is inherited through several generations but only expressed once over-expression is relieved.

There are genetic differences as well as differences in molecular events between the two [*PSI* ^+^] ‘curing’ regimes mediated by wild type and mutant Hsp104, respectively. These are first, that the N-terminal domain of Hsp104 is dispensable for the propagation of all yeast prions, but is required for curing by over-expression (Hung and Masison 2006). Second, the loss of the Hsp90 co-chaperones Sti1 and Cpr7 interferes neither with the propagation of [*PSI* ^+^] nor with the curing of [*PSI* ^+^] by growth in GdnHCl, but loss of either or both does almost abolish the curing by wild type Hsp104 over-expression (Moosavi et al. 2010; Reidy and Masison 2010). Third, an Hsp104 binding site in the M-region of Sup35 allows binding without the cooperation of Hsp70 or Sis1 (Helsen and Glover 2012a, b, Winkler et al. 2012a; Frederick et al. 2014) and deletion of residues 131–140 in the M region of Sup35 eliminates curing by over-expression, but has no other effect on [*PSI* ^+^] propagation (Helsen and Glover 2012a, b) and curing by inhibition of Hsp104 ATPase proceeds normally.

## Hsp104:Sup35 interactions

These genetic differences correlate with different binding regimes of Hsp104 with the Sup35 amyloid substrate: “productive” but labile, leading to prion aggregate fragmentation, presumably common to all Hsp104-dependent yeast prions and “non-productive” and stable, dependent, in Sup35, on Sup35M-domain residues 131–140.

The productive interaction involves Hsp104 being recruited to the substrate by Hsp40 (Sis1)/Hsp70 (Ssa1) chaperones (Tipton et al. 2008; Winkler et al. 2012b; Lee et al. 2013) leading to polymer fragmentation. The second, non-productive binding regime in the M-region of Sup35 allows binding without the cooperation of Hsp70 or Hsp40 but does involve the two Hsp90s, Cpr7 and Sti1 (Helsen and Glover 2012a, b; Winkler et al. 2012a; Frederick et al. 2014). Saarikangas and Barral 2015, have described replicative age-related mother cell retention of, among other proteins, Sup35 [*PSI* ^+^] aggregates. The aggregates involve Hsp104 associated with Hsp70s and are observed in the absence of over-expression of Hsp104. Instability of [*PSI* ^+^] is not observed in these conditions, nor is malpartition. It may be that the association of Sup35 with these objects is the labile one described by Frederick et al. (2014), allowing normal partition and becomes stable with over-expression when the binding involves Hsp90s and not Hsp70s.

Hsp104 has also, apparently two different roles: disaggregation of toxic and misfolded proteins, particularly those resulting from heat-shock, and anchoring misfolded proteins to the actin cytoskeleton. The latter role is associated with cell ageing and involves lantrunculin-sensitive anchoring to the actin cytoskeleton of ageing mother cells as part of the rejuvenation of daughter cells (Tessarz et al. 2009; Helsen and Glover 2012a, b). Ness et al. (2017) propose this latter activity is also likely to be responsible for [*PSI* ^+^] loss induced by over-expression of Hsp104, an activity which is unique to this one prion out of the half-dozen which have been checked.

## Inheritance of [*PSI*^+^] propagons in zygotes and asci

**Fig. 4:**
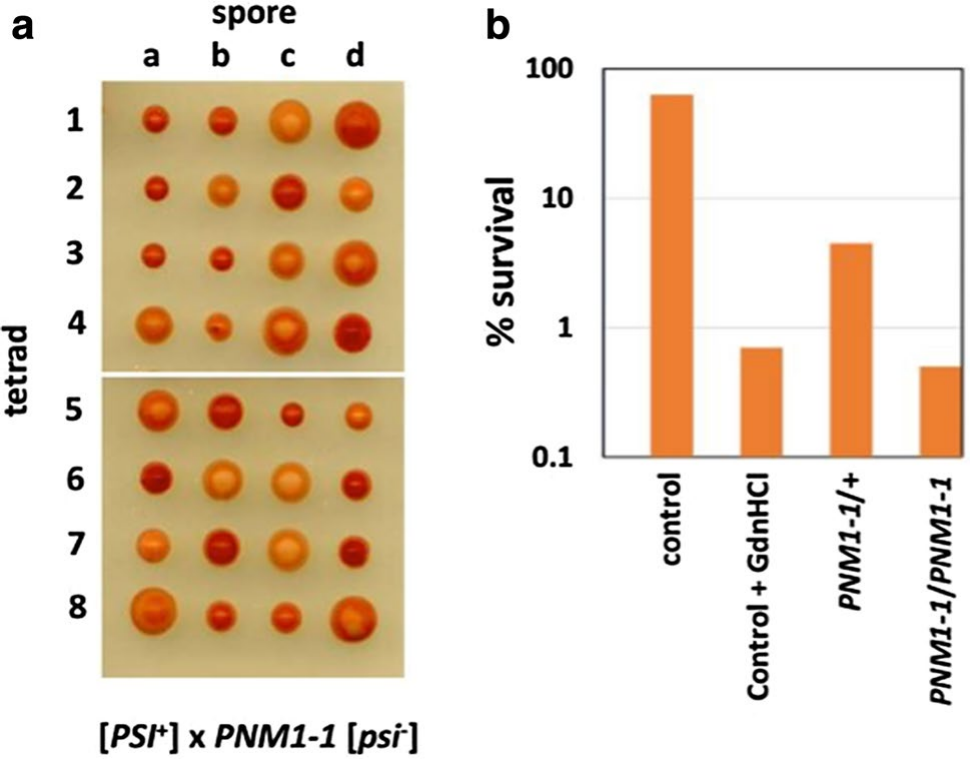
**A** Analysis of the meiotic segregants arising from a *PNM1*-*1* [*psi* ^−^] × [*PSI* ^+^] strain. Each tetrad (numbered *1*–*8*) gives rise to four haploid spore-generated colonies (labelled *a*–*d*). All of the haploid spores are* red* [*psi* ^−^]. **B** Dominance of the *PNM1*-*1* allele of Hsp104 measured by survival of heat shock treatment is illustrated. NB the *x* axis is on a log scale. The survival of WT Hsp104 in the presence of 3 mM guanidine HCl is shown for comparison. The *PNM1*-*1* allele has the following four mutations: F118L, I338T, G619D, G706D (Jones KM, unpublished data) of which the last three are found in the ATPase domains of the enzyme

Hsp104 also has effects, few of which have been reported, on the propagation of the [*PSI* ^+^] prion in sporulation. These effects have been revealed by dominant mutations of Hsp104 which were the first mutants isolated in this gene in 1968 by Hamish Young (Young and Cox 1971). We now call such mutants “PNM” (“Psi No More”) and the two *PNM* loci identified by Young in his original screen are the *HSP104* gene (*PNM1*; Cox, BS, Kerry KM, unpublished) and the *SUP35* gene (*PNM2*; Doel et al. 1994). PNM mutants can prevent the inheritance of [*PSI* ^+^] by spores as shown by the observation that when either *PNM1* [*psi* ^−^] or *PNM2* [*psi* ^−^] mutants are mated with a [*PSI* ^+^] strain the diploids are [*psi* ^−^] and so are all the meiotic products i.e. haploid spores, after sporulation (Fig. 4a). This is not a universal effect because some conformational variants of [*PSI* ^+^] are not eliminated by *PNM1* mutations (Derkatch et al. 1999) consistent with the observation of Frederick et al. (2014) that different conformational variants of Sup35 show differing degrees of Hsp70-independent interaction with Hsp104.

**Fig. 5:**
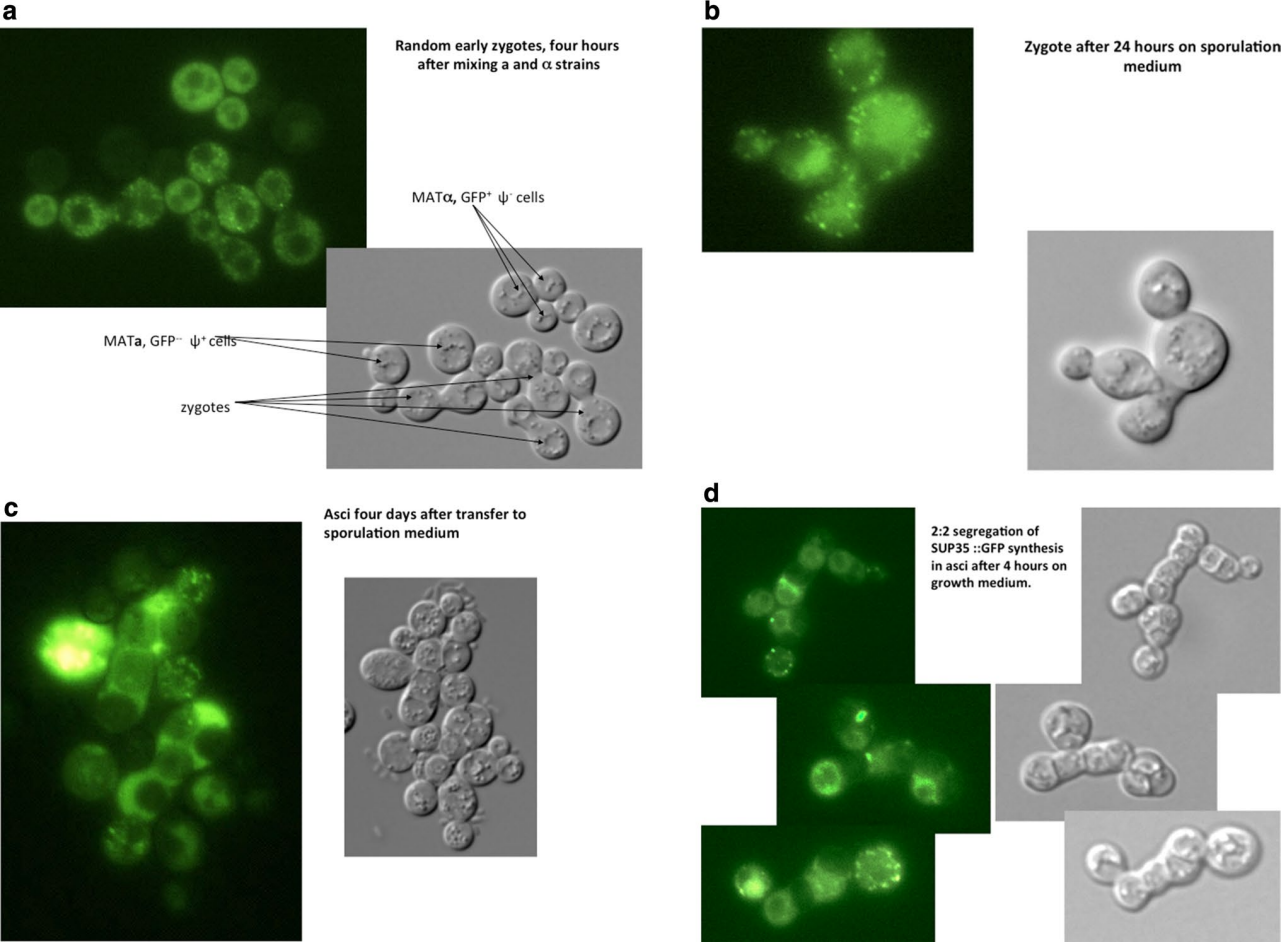
The fate of GFP-decorated [*PSI* ^+^] propagons in sporulation in *PNM1*-*1/*+ [*psi* ^−^] diploids. Fluorescent GFP (*upper panels*) and DIC images (*lower panels*) of cells mating, sporulating and germinating, using the protocols of Satpute-Krishnan et al. (2009). The mating parents were SY81 *MAT*α *PNM1*-*1 SUP35::GFP* (GFP inserted between the M and C domains of Sup35 in the 74D-694 background) [*psi* ^−^] and 74D-694 *MATa* [*PSI* ^+^]. **a** Zygotes formed within 4 h of mixing the parents. The [*PSI* ^+^] status of the *MATa* parent is revealed by the Sup35 prion aggregates being decorated by the Sup35::GFP of the *MATα* parent. **b** An older zygote with buds and GFP aggregates segregating into them, further growth having been arrested 24 h earlier by transfer to sporulation medium. **c** Zygotes with mature spores after four days on sporulation medium. **d** Asci formed in zygotes placed on growth medium for 4 h to promote germination and showing 2:2 segregation of Sup35::GFP synthesis. Little or no trace of the GFP present in the early zygotes is seen in the spores before germination nor after germination, in the two spores in every tetrad that have only the untagged *SUP35* gene. There are very few if any Sup35::GFP-decorated aggregates in the germinating ascospores. The loss of aggregated and diffuse Sup35-GFP is unlikely to be due to Hsp104 disaggregase activity as the *PNM1*-*1* allele has mutations in both ATPase binding domains (Jones KM, unpublished data, see text)

In a *PNM1*.*1* [*psi* ^−^] × [*PSI* ^+^] cross, some of the PNM-induced elimination of [*PSI* ^+^] occurs during the growth of the diploid culture following mating and before sporulation, leading to the 4:0 segregation of [*psi* ^−^]:[*PSI* ^+^] spore cultures (Young and Cox 1971) (Fig. 4a). However, if the switch to sporulation-inducing growth conditions occurs 4 h after the zygote forms and before it can resume growth after mating, more than 90% of the propagons are eliminated in the course of sporulation itself. This effect is illustrated by Fig. 5 and the data in Table 1. (N. B. that the data in this table are taken from a *PNM2* ^*G58R*^ [psi^−^] × [*PSI* ^+^] mating: data from [*psi* ^−^] × [*PNM1*] crosses are not shown but are exactly comparable). Zygotes formed by crossing a *PNM1*.*1* [*psi* ^−^] strain with a ‘strong’ variant of [*PSI* ^+^] 4 h after zygote formation, results in decoration of the propagons from the [*PSI* ^+^] parent with Sup35p from the [*psi* ^−^] cell (e.g. Fig. 5a; Satpute-Krishnan et al. 2009) and an increase in the number of propagons per cell consistent with the increased cell volume of the zygote (Table 1a, b). Analysis of propagon numbers in five tetrads from these zygotes showed a random distribution of 252 propagons per spore with no loss compared with the zygotes’ numbers (Table 1c). By contrast, when the [*psi* ^−^] parent in the cross was replaced with a *PNM2* ^G58R^ [*psi* ^−^] strain and the same sporulation regime applied (Table 1d), 60% of the spores had no propagons with the total number of propagons in the 20 spores scored being 4% of the total number of propagons scored in the five tetrads with the wild-type [*psi* ^−^] diploid. It should be noted that half the spores analysed in this cross would carry the *PNM2* gene so any propagons they inherited would not be detectable by the assay we employ. Figure 5c, d appear to corroborate exactly these genetic data, including the 2:2 segregation of the PNM genes involved, apparent on germination.

**Table 1:** The effect on the inheritance of [*PSI* ^+^] propagons in zygotes and asci from matings of (a), MATa, [*PSI* ^+^] with (b and c) MATα [*psi* ^−^] or (d) with PNM2(Sup35^G58R^) [psi^−^] haploids

(a) ψ+ strong parent
74, 422, 185, 486, 504, 205, 670, 215, 440, 158, 284, 317, 408, 275, 390, 265, 331, 413, 393, 239, 370, 315, 370, 783
Ave.: 382.5; median: 324
(b) 4-h Zygotes (ψ+ × ψ−)
1316, 2600, 612, 798, 529, 642, 504, 1414, 464, 780, 953, 248, 652, 1081, 178, 698, 682, 1749, 2176, 736, 299
Ave.: 848; median: 698

(c) ψ- x ψ+, Propagons in five tetrads from 4-h-old zygotes					
Ascus	1	2	3	4	5
Spore: a	159	225	293	150	209
b	192	232	264	106	234
c	197	195	331	397	158
d	373	–	532	261	282
Total	921	–	1420	914	883
Ave.	260.5	217	305	228.5	220.75
Total overall: 4790; Ave./spore 252.1					

(d) G58R x ψ+, Propagons in five tetrads from 4-h-old zygotes					
Ascus	1	2	3	4	5
Spore: a	7	0	0	0	0
b	9	0	17	0	45
c	0	2	0	88	0
d	0	23	0	0	6
Total overall: 191; Ave./spore: 9.05 (19.7 in WT spores)					

Propagons in (a) [*PSI* ^+^] haploid cells; (b) in zygotes formed 4 h after mating this [*PSI* ^+^] haploid with a PNM2(Sup35^G58R^) [*psi* ^−^] haploid; (c) in spores in tetrads from 4-h-old zygotes of this mating and (d) in spores formed in 4-h-old zygotes of the [*PSI* ^+^] parent mated with PNM2.^G58R^ ([*psi* ^−^]), all derived from 74D694 [*PSI* ^+^]. Propagons were counted in (b) 4-h-old zygotes, in (a) haploid [*PSI* ^+^] parent unbudded cells and (c, d) in spores from asci formed from 4-h-old zygotes placed on 1% acetate medium. Single unbudded zygotes, haploid unbudded cells and spores from tetrads were picked by micromanipulation to form colonies on rich growth medium (YEPD) 3 mM in guanidine HCl to inhibit the ATPase of Hsp104, to count propagons by the single-cell method method of Cox et al. (2003). Note that in some zygotes (b), doubling of propagon numbers seems not to have occurred before they were picked, but there is no evidence of a failure in doubling in any of the five tetrads sampled. Compare with Fig. 5

## Hsp104 and transmission of [*PSI*^+^] propagons in sporulation

The cross discussed above (Table 1) involved a PNM allele of *SUP35* (i.e. *PNM2* ^G58R^) not *HSP104* which leaves open the question whether the elimination of [*PSI* ^+^] in this cross is due to an unusual sensitivity of the mutant protein to Hsp104 disaggregase. However, we find that dominant *PNM1* mutants of Hsp104 totally lacking ATPase activity give results either quantitatively indistinguishable from those in Table 1, or are even more severe in terms of reduction of propagon numbers (Cox, BS unpublished data: see below).

Loss of [*PSI* ^+^] in the *PNM2* ^*G58R*^ [*psi* ^−^] × [*PSI* ^+^] would not appear to be an amyloid disaggregation problem, but a propagon segregation problem. Nevertheless, in the absence of any perturbation of Hsp104 function by mutation or environment, all the empirical evidence is consistent with a random distribution of [*PSI* ^+^] propagons in both vegetative (mitotic) and sporulation (meiotic) divisions (Table 1; Byrne et al. 2009). Mutations or environmental disturbance may nevertheless affect inheritance in either.

The role of Hsp104 in sporulation has not been explored very extensively although sporulation occurs normally in diploids homozygous for a *∆hsp104* deletion (e.g. Ünal et al. 2011). The possibility remains that, without being essential Hsp104 nevertheless has a role which is important for the distribution of [*PSI* ^+^] propagons and perhaps other organelles to spores (see Suda et al. 2007). The possibility that a link between these observations and the rejuvenation which accompanies spore formation (Ünal and Amon 2011; Ünal et al. 2011) is tantalizing. To explore this we followed the fate of GFP-decorated propagons in sporulation in *PNM1*-*1/*+ [*psi* ^−^] diploids. This diploid segregated an average of seven propagons to spores in a total of 10 tetrads analysed. In spite of a starting cytoplasm replete with diffuse GFP and GFP-decorated punctate spots before the arrest of vegetative division and also for 24 h after the induction of meiosis (Fig. 5a, b), by the time sporulation was complete after four days, asci showed no fluorescence within the four spores and only weak diffuse fluorescence outside them (Fig. 5c). Once returned to growth medium, GFP fluorescence reappeared in the two spores in every ascus to which the Sup35::GFP fusion gene segregated, mostly without the evidence of Sup35::GFP-decorated aggregates (Fig. 5d) and there was minimal evidence of GFP-decorated aggregates inherited from the zygotes in which these ascospores formed. In the presence of the dominant *PNM1*-*1* mutation in these sporulating zygotes we estimate that at most only 10% of the Sup35, whether aggregated or not, was dispersed to the spores. What we see is Mendelian inheritance of autonomous new synthesis of this GFP-linked essential protein in the germinating spores, very little of which decorates any Sup35 aggregates.

## Degradation, disaggregation or malpartition?

Another question we raise in our new study (Ness et al. 2017) is that of the source of instability of the [*PSI* ^+^] prion that is commonly observed in [*PSI* ^+^] variants, *PNM2(SUP35)* mutants and heterozygotes formed between them and with strains expressing wild type Sup35, and also in partially dominant *PNM1*(*HSP104*) mutants. Such prion instability characteristically appears as sectoring in colonies growing on normal growth medium (Fig. 1c) and the implication is that the instability is associated with malpartition rather than degradation. No doubt having very low numbers of large propagons would contribute to the spectrum of instability, but interaction with Hsp104 in rejuvenation mode may be a common feature (Verges et al. 2011).

What is not certain is that any form of intrinsic or induced instability of Sup35/[*PSI* ^+^] prions is due to significant degradation or disaggregation of the amyloid form. Disaggregation can be observed in vitro (Shorter and Lindquist 2004) albeit with molar ratios of Hsp104:Sup35 polymer that are far in excess of what may be observed in vivo, but until now (i.e. Ness et al. 2017) has not been directly assayed in vivo.

One of the epidemiological myths about the mammalian PrP^Sc^ prion is that it is indestructible by normal physical or chemical methods. For example, sterilizing surgical instruments is not sufficient to quell iatrogenic prion infection, nor is cooking; resistance to proteases has been used as a means of identifying amyloid and Alper et al. (1967) could not kill the infectious scrapie agent by ionizing radiation or UV. The thermodynamic facts are that an amyloid fibre exists in an entropy pit and relatively large amounts of energy input are required to get it out (Eichner and Radford 2011). Fragmentation by Hsp104 with the 12 ATPase sites in the hexamolecular collar is achieved by merely extracting a single molecule from within the fibre (Glover and Lum 2009). In spite of several published claims to the contrary, there has been no direct demonstration of in vivo degradation of [*PSI* ^+^] Sup35 amyloid aggregates. This leaves us with the need to find other explanations for the appearance of prion-free cells.
